# Neoadjuvant treatment for resectable pancreatic adenocarcinoma: What is the best protocol?

**DOI:** 10.1002/ags3.12311

**Published:** 2020-02-18

**Authors:** Fuyuhiko Motoi, Michiaki Unno

**Affiliations:** ^1^ Department of Surgery Tohoku University Graduate School of Medicine Aoba‐ku Japan

**Keywords:** neoadjuvant chemotherapy, neoadjuvant therapy, pancreatic adenocarcinoma, resectable pancreatic cancer

## Abstract

Although upfront surgery has been the gold standard for pancreatic adenocarcinoma that is planned for resection, it should be compared with the alternative strategy of neoadjuvant therapy. Despite the many reports of the efficacy of neoadjuvant therapy, most of them were not comparative. Recently Prep‐02/JSAP05 study clearly demonstrated the significant survival benefit of neoadjuvant chemotherapy over upfront surgery for pancreatic adenocarcinoma that is planned for resection. These findings opened a new chapter of neoadjuvant therapy. Ongoing trials are expected to confirm the evidence. This review summarizes the past, present, and future perspectives of neoadjuvant therapy and its optimization.

## Graphical abstract

Recently, Prep‐02/JSAP05 study demonstrated the significant survival benefit of neoadjuvant chemotherapy over upfront surgery for pancreatic adenocarcinoma that is planned for resection. This review summarizes the past, present, and future perspectives of neoadjuvant and its optimization.

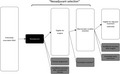

## 1 RESECTABILITY OF PANCREATIC ADENOCARCINOMA

Several definitions of resectability of pancreatic adenocarcinoma (PDAC) have been approved for determining the possibility for complete clearance (R0 resection) by surgery, taking into account oncological and general aspects.^1^, ^2^, ^3^, ^4^ Surgical resectability of PDAC is assessed by the evaluation of local tumor extension to vessels and distant metastases. Excluding tumor with distant metastases, which is defined as unresectable with metastases (UR‐M), local resectability is classified in three categories: resectable (R), borderline resectable (BR), and unresectable (UR‐LA). R PDAC shows no vascular infiltration to major vessels. Complete clearance of R tumor is required in standard pancreatectomy without combined vascular resection. BR PDAC is sub‐classified into two categories: BR‐PV showing PV distortion or narrowing, and BR‐A showing semi‐circumferential abutment with a major artery. There is a theoretical “borderline” between BR‐PV and BR‐A. Whereas PV resection is currently recommended for achieving R0 resection,^5^, ^6^ arterial resection remains controversial due to significantly increased rates of morbidity.^6^ From the surgical perspective, BR‐PV PDAC is borderline resectable, whereas BR‐A PDAC is borderline unresectable. Considering surgical feasibility, R and BR‐PV PDAC should be considered as candidates for “PDAC that is planned for resection (potentially resectable PDAC).” Potentially resectable PDAC has been treated by upfront surgery,^1^, ^2^ although neoadjuvant for BR PDAC might be considered given the poor oncological outcomes.^7^

## 2 POTENTIALLY RESECTABLE PDAC

**Figure 1:**
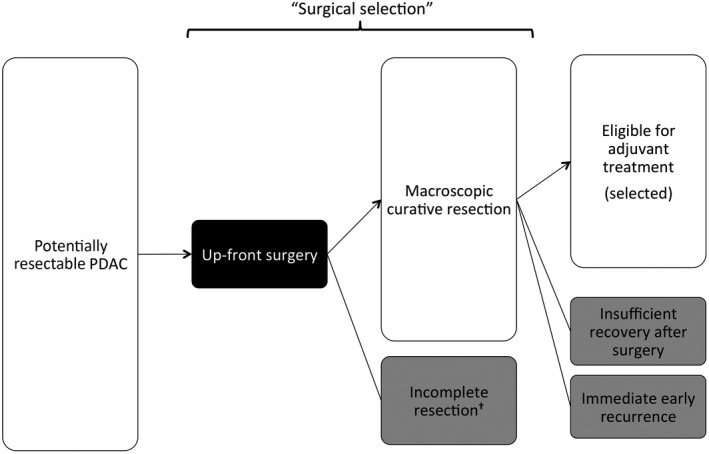
Patient selection for up‐front surgical strategy. The gray box represents ineligible cases for postoperative adjuvant treatment. ^†^Incomplete resection includes unresectable at the time of surgery, macroscopic positive margin resection (R2 resection), resection with metastatic disease (M1)

Upfront surgery has been the gold standard for potentially resectable PDAC, as well as for most other solid cancers. Adjuvant therapy (adjuvant) is administered for macroscopically curatively resected PDAC with full recovery in the planned postoperative period and without immediate early recurrence (Figure 1). This cohort benefits from recent advances of adjuvant chemotherapy. Randomized controlled trials (RCTs) of adjuvant chemotherapy reported that the median overall survival (OS) these selected patients reached was 46.5 months with S1 adjuvant^8^ and 54.4 months with modified FOLFIRINOX adjuvant.^9^ Adjuvant for patients with resected PDAC, who are eligible after selection for surgery (Figure 1), is fully accepted as the standard based on solid evidence. In contrast to eligible patients, patients with aggressive tumor (incompletely resectable,^10^ immediately recurred,^11^ or vulnerable for treatment (insufficiently recovered^12^) who show a poor prognosis are excluded from analysis. Unfortunately, it is hard to discriminate, before surgery, between eligible patients and ineligible patients for adjuvant. Since potentially resectable PDAC is not equal to resected PDAC eligible for adjuvant (Figure 1), it is not convincing that upfront surgery is the optimal strategy for potentially resectable PDAC. The optimal strategy should be explored by a comparison between upfront surgery and the alternative strategy of neoadjuvant therapy (neoadjuvant) followed by surgery.

## 3 PROSPECTIVE STUDIES AND META‐ANALYSES

**Figure 2:**
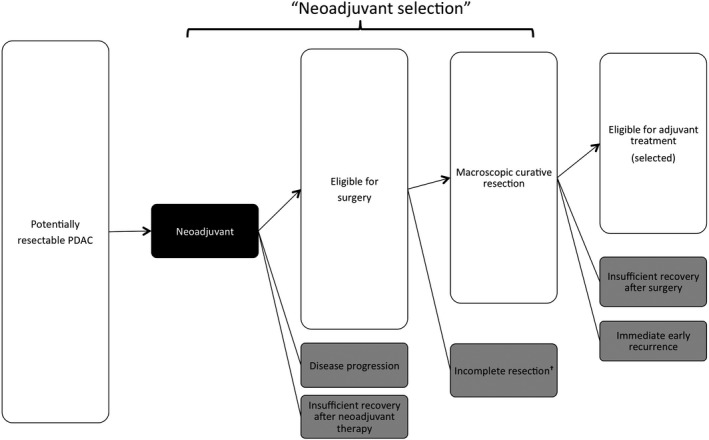
Patient selection for neoadjuvant strategy. The gray box represents ineligible cases for postoperative adjuvant treatment. ^†^Incomplete resection includes unresectable at the time of surgery, macroscopic positive margin resection (R2 resection), resection with metastatic disease (M1)

As well as upfront surgery, neoadjuvant followed by surgery has patient selection during the neoadjuvant period, in addition to surgical selection (Figure 2). In retrospective or case‐series studies of neoadjuvant for PDAC, the survival outcome of only resected PDAC after neoadjuvant appeared to show a theoretically better trend because of exclusion of patients with a poor prognosis. This selection usually causes significant bias^13^ even in large‐scale studies. In contrast to retrospective analyses, a prospectively designed interventional study can provide low‐biased survival data by intention‐to‐treat (ITT) analysis. Several prospective studies of neoadjuvant reported survival outcomes including data from ITT analyses. Talamonti et al^14^ reported the results of a multi‐institutional phase II trial of neoadjuvant chemoradiotherapy (NACRT), demonstrating a high rate of negative margin resection and no nodal involvement. Mornex et al^15^ described the feasibility and efficacy of NACRT as acceptable feasibility. Palmer et al conducted a randomized phase II trial comparing the regimens of neoadjuvant chemotherapy (NAC). They showed the superiority of combination therapy to monotherapy, with high resection and survival rates.^16^ Heinrich et al^17^ also reported the safety and effect of NAC with a similar regimen associated with improved quality of life and nutritional status. The survival outcome of NACRT with a combination regimen and that with monotherapy suggested that the combination regimen did not improve the outcome.^18^, ^19^ Landry et al^20^ conducted a randomized phase II study of NACRT comparing induction chemotherapy followed by NACRT. Although the resection rate was low compared to other studies, the survival of resected cases was comparable. Turrini et al^21^ reported the results of their phase II study of NACRT. Pipas et al^22^ reported a single‐institutional phase II study of NACRT. Motoi et al^23^ conducted a prospective phase II trial of NAC with gemcitabine plus S1 (NAC‐GS) in a multi‐institutional setting. NAC‐GS was well‐tolerated, with a good survival outcome without radiotherapy. OʼReilly et al^24^ reported the results of their phase II study of NAC with a combination regimen, suggesting a longer survival rate in both the ITT cohort and the cases who underwent resection. Okano et al^25^ published the data from a trial of NACRT demonstrating high survival rates. Motoi et al^26^ again reported a large‐scale phase II trial of NAC‐GS in another multi‐centre setting with a reproducible survival outcome. Tsai et al^27^ reported the results of a phase II trial of neoadjuvant based on molecular profiling. Eguchi et al^28^ reported a good outcome from a phase II trial of NACRT with a GS regimen. These results are summarized in Table 1.^14^, ^15^, ^16^, ^17^, ^18^, ^19^, ^20^, ^21^, ^22^, ^23^, ^24^, ^25^, ^26^, ^27^, ^28^ Although these studies reported the survival outcome of an ITT cohort, none of them had a cohort treated by upfront surgery as a control.^14^, ^15^, ^16^, ^17^, ^18^, ^19^, ^20^, ^21^, ^22^, ^23^, ^24^, ^25^, ^26^, ^27^, ^28^

**Table 1:** Prospective phase II trials of neoadjuvant therapies for resectable pancreatic cancer

Author	N	Inclusion	Modality and Regimen of Neoadjuvant Intervention	Resection rate	OS (ITT)	OS (resected)	Reference
Talamonti MS	20	R, BR	GEM + RT	85%	18	26	14
Mornex F	41	R, BR	5FU/CIS + RT	63%	9.4	11.7	15
Palmer DH	50	R, BR	GEM (n = 24) GEM/CIS (n = 26)	38% 70%	9.9 15.6	28.4	16
Heinrich S	28	R	GEM/CIS	80%	26.5	19.1	17
Varadhachary GR	90	R	GEM/CIS + RT	66%	17.4	31	18
Evans DB	86	R	GEM + RT	74%	22.7	34	19
Landry J	21	R, BR	GEM + RT (n = 10) GEM/CIS/5FU → 5FU+RT (n = 11)	24%	19.4 13,4	26.3	20
Turrini O	34	R	DOC + RT	50%	15.5	31.9	21
Pipas JM	37	R, BR	GEM/CET + RT	76%	17.3	24.3	22
Motoi F	36	R, BR	GEM/S1	87%	19.7	34.7	23
OʼReilly EM	38	R	GEM/OX	71%	27.2	N. R.	24
Okano K	57	R, BR	S1 + RT	91%	N. R.	N. R.	25
Motoi F	101	R, BR	GEM/S1	73%	30.8	N. R.	26
Tsai S	130	R, BR	FOLFIRINOX (n = 52) FOLFIRI (n = 26) GEM/Nab‐P (n = 16) CAP/Nab‐P (n = 15) *+RT (n = 83)	82%	38	45	27
Eguchi H	63	R	GEM/S1 + RT	86%	55.3	NR	28

Abbreviations: 5FU, 5‐fluorouracil; BR, Borderline Resectable; CAP, Capecitabine; CET, Cetuximab; CIS, Cisplatin; DOC, Docetaxel; GEM, Gemcitabine; N, Number in the cohort; NR, did not reach the median time; Nab‐P, Nab‐Paclitaxel; OS (ITT), median overall survival in months by intention‐to‐treat analysis; OS (resected), median overall survival in months for resected cases; OX, Oxaliplatin; R, Resectable; RT, Radiation.

Several meta‐analyses investigated the efficacy of neoadjuvant.^29^, ^30^, ^31^, ^32^ D'Angelo et al^29^ summarized the survival outcome of 12 neoadjuvant studies including 628 patients. The estimated median OS rate of the ITT cohort and the resected cohort were 16.7 months and 22.78 months, respectively. Dhi et al^30^ reviewed 5520 patients from 96 studies in a meta‐analysis. They reported that the estimated resection rate of neoadjuvant for R PDAC was 80%. Both reviews analysed only neoadjuvant studies, with no comparison to upfront surgery. Versteijne et al^31^ compared neoadjuvant with upfront surgery in a meta‐analysis. They included only the 38 studies that reported survival data by ITT analysis. The weighted median OS by ITT was 18.8 months in the neoadjuvant patients and 14.8 months in the upfront surgery patients. Unno also compared the survival outcomes of neoadjuvant with upfront surgery in a meta‐analysis including only ITT data.^32^ The results of the meta‐analysis showed that the patients treated with neoadjuvant had better long‐term survival than those treated with upfront surgery. Though these analyses demonstrated the improvement of survival by neoadjuvant, the results were not conclusive.

## 4 RANDOMIZED, CONTROLLED TRIALS

Unfortunately, limited reports of RCTs of neoadjuvant compared with upfront surgery have been published. Golcher et al^33^ reported the first RCT of NACRT compared with upfront surgery. Although their neoadjuvant intervention was feasible, the trial was terminated early due to slow recruitment and the results were not significant. Casadei et al^34^ reported an RCT of NACRT. They also did not show a significant difference between the arms due to difficulty recruiting patients. Jang et al published the results of an RCT of NACRT limited to BR PDAC.^35^ Although the number of cases was small, which raised some criticism of their study design and conduct,^7^ they showed significant oncological benefits of NACRT for BR PDAC compared with upfront surgery. Van Tienhoven et al^36^ conducted an RCT of NACRT for R and BR PDAC (PREOPANC‐1 trial). Their preliminary results showed an improved trend in OS by NACRT, but it was not significant.^37^ Several secondary endpoints were shown to be in favour of NACRT, including the R0 resection rate and disease‐free survival.

Unno and Motoi et al^38^ conducted an RCT of NAC‐GS (Prep‐02/JSAP05). A total of 362 patients with R or BR‐PV PDAC were randomly assigned to NAC‐GS or upfront surgery. The median OS was 36.7 months for NAC‐GS and 26.6 months for upfront surgery (*P* = .015).^39^, ^40^ The resection rates of both arms were similar, with no operative mortality. A significant decrease of pathological nodal metastases and hepatic relapse after surgery was noted in the NAC‐GS patients compared to upfront surgery patients.^40^ Based on the results of this adequately powered RCT, it was concluded that the strategy of NAC‐GS could be a new standard for potentially resectable PDAC. These data from RCTs are summarized in Table 2. There were differences among the five trials, including types of intervention and eligibility for the study. Although the survival outcomes of these trials were different, the resection rates for upfront surgery were comparable, ranging from 70% to 78%. The resection rate of NACRT, which was quite similar and ranged from 61% to 63%,^33^, ^34^, ^35^, ^37^ was about 10% lower than that of each control. Only selected cases after neoadjuvant might benefit from NACRT intervention with its advantage for local treatment, as suggested by the PREOPANC‐1 trial.^37^ The resection rate after NAC did not decrease compared to that of control upfront surgery.^39^, ^40^ In contrast to NACRT, potentially resectable PDAC could benefit from NAC due to its nature as a systemic treatment.

**Table 2:** Randomized, controlled trials for resectable or borderline resectable pancreatic cancer comparing neoadjuvant intervention with up‐front surgery

Author	Inclusion	Modality and regimens of neoadjuvant therapy	N	Resection rate	OS (ITT)	Hazard ratio	*P* value	Reference
Golcher H	R	GEM/CIS + RT	30	63%	17.4	‐	.96	33
		Up‐front surgery	33	70%	14.4			
Casadei R	R	GEM + RT	18	61%	22.4	‐	.97	34
		Up‐front surgery	20	75%	19.5			
Jang JY	BR	GEM + RT	27	63%	21	0.51	.028	35
		Up‐front surgery	23	78%	12			
Van Tienhoven GSM	R, BR	GEM + RT	119	62%	17.1	0.74	.074	36, 37
		Up‐front surgery	127	72%	13.7			
Unno M	R, BR(‐PV)	GEM/S1	182	77%	36.72	0.72	.015	38, 39, 40
		Up‐front surgery	180	72%	26.65			
Heinrich S	R	GEM/OX	155	Ongoing (results not yet reported)				41
		Up‐front surgery	155					
Tachezy M	BR	GEM + RT	205	Ongoing (results not yet reported)				42
		Up‐front surgery	205					
Labori KJ	R	FOLFIRINOX	54	Ongoing (results not yet reported)				43
		Up‐front surgery	36					
Schwarz L	R	FOLFIRINOX	64	Ongoing (results not yet reported)				44
		FOLFOX	64					
		Up‐front surgery	32					

Abbreviations: BR(‐PV), borderline resectable with portal vein invasion; BR, borderline resectable; CIS, cisplatin; GEM, gemcitabine; N, number in the cohort; OS (ITT), median overall survival in months by intention‐to‐treat analysis; OX, oxaliplatin; R, resectable; RT, radiation.

Several RCTs of neoadjuvant compared with upfront surgery as the control have been ongoing. Heinrich et al^41^ conducted a trial comparing NAC to upfront surgery (NEOPAC). Tachezy et al^42^ planned the NEOPA study to compare NACRT with upfront surgery for BR PDAC. Labori et al^43^ conducted a trial using NAC compared with upfront surgery (NorPACT‐1). In addition to the final results from the PREOPANC‐1 trial,^36^, ^37^ the results from ongoing trials added information regarding neoadjuvant (Table 2).

## 5 OPTIMAL PROTOCOL FOR NEOADJUVANT THERAPY

Two cycles of the GS regimen, which was used in the Prep‐02/JSAP05 study, have been a standard regimen for NAC, at least in Japan, for potentially resectable PDAC.^38^, ^39^, ^40^ Although several prospective trials using other regimens, which include radiotherapy, are ongoing, their results have yet been clearly reported.^36^, ^37^, ^41^, ^42^, ^43^, ^44^ Considering recent progress in chemotherapy for UR PDAC,^45^, ^46^ a clinical question has been raised about the optimal protocol in the neoadjuvant setting.

## 6 THE OPTIMAL REGIMEN FOR NEOADJUVANT CHEMOTHERAPY

Two major regimens for UR PDAC have been provided as standard based on the results of RCTs. Conroy et al^45^ first demonstrated the superiority of combination chemotherapy FOLFIRINOX compared to single‐agent gemcitabine. Von Hoff et al^46^ also reported the superiority of another combination regimen, gemcitabine plus nab‐paclitaxel, compared to single‐agent gemcitabine. These regimens would be strong candidates for the optimal regimen in the neoadjuvant setting. In the treatment of UR PDAC without surgery, significant improvement of OS is the most important outcome. In the neoadjuvant setting, where tumor would be resected after a certain period of neoadjuvant, improvement of both the response rate and progression‐free survival (PFS) might be of importance. Three other studies, which failed to show a longer OS than single‐agent control, but showed a higher response rate and longer PFS, should be picked up in addition to two standard regimens. Ueno et al reported that the GS regimen, which was used in the Prep‐02/JSAP05 study and demonstrated positive results,^39^, ^40^ showed a significantly higher response rate and longer PFS than gemcitabine single‐agent.^47^ Ozaka et al^48^ also reported similar results, with a high response rate and longer PFS, in a randomized, phase II trial. Louvet et al^41^ reported that gemcitabine and oxaliplatin, which were used in a neoadjuvant study, showed a significantly higher response rate and longer PFS than gemcitabine alone.^49^ Cunningham et al^50^ also demonstrated a significantly higher response rate and longer PFS with combination gemcitabine plus capecitabine than with gemcitabine alone. These combination regimens might be candidates for the optimal NAC regimen, and they are summarized in Table 3.

**Table 3:** Randomized, controlled trials of chemotherapy for unresectable pancreatic cancer

Author	Arm	N	Response rate	*P* value	PFS	*P* value	OS	*P* value	Reference
Conroy T	FOLFIRINOX	119	31.6%	<.001	6.4	<.001	11.1	<.001	45
	GEM	127	9.4%		3.3		6.8		
Von Hoff DD	GEM/Nab‐P	182	29%	<.001	5.5	<.001	8.5	<.001	46
	GEM	180	8%		3.7		6.7		
Ueno H	GEM/S1	275	29.3%	<.001	5.7	<.001	10.1	.15	47
	GEM	277	13.3%		4.1		8.8		
Louvet C	GEM/OX	157	26.8%	.04	5.8	.04	9.0	.13	49
	GEM	155	17.3%		3.7		7.1		
Cunningham D	GEM/CAP	267	19.1%	.03	5.3	.004	7.1	.08	50
	GEM	266	12.4%		3.8		6.2		

Abbreviations: CAP, Capecitabine; GEM, Gemcitabine; N, Number in the cohort; Nab‐P, Nab‐Paclitaxel; OS, median overall survival in months of each arm; OX, Oxaliplatin; PFS, median progression‐free survival in months of each arm.

In the adjuvant setting, the modified FOLFIRINOX was more active than gemcitabine.^9^ In a similar setting, however, gemcitabine plus nab‐paclitaxel showed marginal results that were not significant with respect to recurrence‐free survival compared to gemcitabine.^51^ The trial comparing gemcitabine plus capecitabine to gemcitabine in the adjuvant setting (ESPAC‐4) showed positive results.^52^ Murakami et al^53^ reported, in a retrospective analysis, that the GS regimen was active in the adjuvant setting. Given the efficacy of FOLFIRINOX in various settings,^9^, ^45^ this regimen would be one of the most attractive candidates to be evaluated as NAC, compared with NAC‐GS as a control. In any case, a well‐designed RCT is necessary to explore the optimal regimen.

## 7 CHEMOTHERAPY AND/OR RADIOTHERAPY?

Chemoradiotherapy is an attractive modality in the treatment of PDAC for local disease control. In the neoadjuvant setting, an increase of R0 resection, which would decrease local relapse after resection, is expected for NACRT. Many prospective non‐randomized trials of NACRT have been reported.^14^, ^15^, ^18^, ^19^, ^20^, ^21^, ^22^, ^25^, ^27^, ^28^ RCTs comparing NACRT with upfront surgery, however, have not yet been fully reported,^33^, ^34^, ^35^, ^36^, ^37^ in contrast to NAC.^38^, ^39^, ^40^ The reduction of hepatic relapse after surgery with NAC^40^ might suggest that pre‐operative systemic delivery of combination chemotherapy would be necessary to impede the progression of micrometastases even in R PDAC, providing long‐term survival. The low resection rate of NACRT^33^, ^34^, ^37^ might be partly because previous studies used a reduced dose of systemic chemotherapy in combination with radiotherapy. Katz et al^54^ reported the feasibility and efficacy of NAC FOLFIRINOX followed by NACRT for BR PDAC in multi‐institutional trials. Murphy et al reported the results of longer NAC FOLFIRINOX followed by radiotherapy for BR PDAC, with good survival outcomes.^55^ Their concept is “total neoadjuvant therapy (TNT),” an emerging approach with excellent outcomes for other cancers.^56^ The group also reported the effect of TNT for UR PDAC, with excellent survival.^57^ These strategies might also improve the survival outcome of R PDAC. The use of radiation should be examined, rather than assuming an either/or scenario in a prospective trial.

## 8 OPTIMAL DURATION FOR NEOADJUVANT

The duration of neoadjuvant was about 2 months in most previously reported trials for R PDAC.^2^, ^24^, ^26^, ^28^, ^38^ A longer course of neoadjuvant might improve the survival outcome when the regimen continues to be active after the initial treatment period of 2 months. Excluding non‐responders^10^, ^11^ and also vulnerable cases,^12^ the addition of effective treatment would have a good effect on tumor control. Compared to other types of cancer that are chemotherapy‐ or radiotherapy‐sensitive, pathological complete response (CR) is rarely obtained after neoadjuvant for PDAC.^25^, ^28^ Though two cycles of NAC‐GS showed significant survival benefit for potentially resectable PDAC,^39^, ^40^ it might not be of sufficient duration for a large proportion of PDAC patients because of its poor prognosis.

For neoadjuvant for UR and BR PDAC including the concept of “conversion surgery,” several reports demonstrated longer duration of the treatment before surgery.^58^, ^59^, ^60^ Longer duration of neoadjuvant would be necessary; therefore, an accurate assessment of response using appropriate surrogate markers to decide on treatment continuation would be essential to avoid detrimental elongation of the treatment.

## 9 SURROGATE ENDPOINT FOR NEOADJUVANT

To compare many types of interventions, surrogate endpoints are needed to select the optimal intervention. Surrogate endpoints for OS have not been established for PDAC in the neoadjuvant setting. Although the R0 resection rate has been considered to be the main goal for cancer surgery, it might only be a minimal requirement for long‐term survival. Actually, no significant difference of the R0 resection rate between NAC and upfront surgery was observed in the Prep‐02/JSAP05 trial, where a significant difference in OS was observed.^39^, ^40^ Since R0 resection could reflect only local clearance of the tumor, it would not be a suitable surrogate endpoint for OS in PDAC, which is systemic disease even in R PDAC.

The pathological effect after neoadjuvant might be another candidate surrogate endpoint for OS. Pathological CR following neoadjuvant has been shown to be associated with long‐term survival in other types of cancer, including breast^61^ and rectum.^62^ In neoadjuvant for PDAC, however, pathological CR is rarely obtained even after multi‐modal treatment.^25^, ^28^, ^63^ Although a good pathological effect after neoadjuvant would be presumed to lead to longer survival,^64^ it remains to be elucidated in the neoadjuvant setting of PDAC.

Radiological response would be a candidate surrogate endpoint. Radiological assessment, which could be performed before surgery, is superior to pathological assessment in clinical decision‐making. Radiological CR could reflect pathological CR, which could be a surrogate endpoint of survival in the other types of cancer described above. As well as pathological CR, radiological CR of PDAC is rarely obtained even after multi‐modal treatment.^28^, ^55^

Serum tumor markers and their kinetics are other promising candidates as surrogate endpoints. CA19‐9, which is increased in most PDACs at baseline, is widely used as a tumor marker. A decrease of tumor markers after therapy reflects a good response and longer survival for responders.^64^ For resected PDAC, a decrease of CA19‐9 to the normal range after surgery is associated with longer survival and a low hepatic relapse rate.^65^, ^66^ The CA19‐9 level, which can be measured less‐invasively and quantitatively, has several advantages as a surrogate endpoint. In a proportion of the cases with normal CA19‐9 levels after surgery following neoadjuvant, CA19‐9 levels would be a surrogate endpoint of survival to select an optimal regimen or duration of the treatment. However, further efforts are still needed to determine the optimal cut‐off point of tumor marker as a surrogate endpoint.

## 10 CONCLUSION

Recently evidence opened a new chapter of the neoadjuvant era for PDAC. However, it was only a beginning, and further efforts are needed to optimize it with adequate surrogate markers.

## DISCLOSURE

Conflict of Interest: Author FM and MU received lecture fee from Taiho Pharmaceutical. Author MU was supported by grant from Takeda Pharmaceutical Company.
